# Benchmarking Based on Regularly Recorded Claw Health Data of Austrian Dairy Cattle for Implementation in the Cattle Data Network (RDV)

**DOI:** 10.3390/ani12070808

**Published:** 2022-03-22

**Authors:** Johann Kofler, Marlene Suntinger, Martin Mayerhofer, Kristina Linke, Lorenz Maurer, Alexandra Hund, Andrea Fiedler, Jürgen Duda, Christa Egger-Danner

**Affiliations:** 1 Department of Farm Animals and Veterinary Public Health, University Clinic for Ruminants, University of Veterinary Medicine Vienna, 1210 Vienna, Austria; alexandra.hund@lazbw.bwl.de; 2 ZuchtData EDV-Dienstleistungen GmbH, 1200 Vienna, Austria; suntinger@zuchtdata.at (M.S.); mayerhofer@zuchtdata.at (M.M.); linke@zuchtdata.at (K.L.); egger-danner@zuchtdata.at (C.E.-D.); 3 Department of Sustainable Agricultural Systems, University of Natural Resources and Livestock Sciences, 1180 Vienna, Austria; lorenz.maurer@boku.ac.at; 4 Landwirtschaftliches Zentrum für Rinderhaltung, Grünlandwirtschaft, Milchwirtschaft, Wild und Fischerei Baden-Württemberg (LAZBW), 88326 Aulendorf, Germany; 5 Praxisgemeinschaft für Klauengesundheit, 81247 Munich, Germany; fiedler@praxis-klauengesundheit.de; 6 Landeskuratorium der Erzeugerringe für Tierische Veredelung in Bayern e.V. (LKV), 80687 München, Germany; juergen.duda@lkv.bayern.de

**Keywords:** lameness, claw lesions, ‘alarm’ lesions, claw trimming, electronic recording, culling rate, cattle, benchmarking

## Abstract

**Simple Summary:**

Benchmarking is an assessment process that compares an individual entity to their peer group and the ‘best in class’. The goal is to motivate for improvement, e.g., claw health in dairy farms. We describe the pre-requisites necessary for establishing a benchmarking system for claw health. Data were transmitted by hoof trimmers, who recorded claw lesions of dairy cows in 512 herds during each trimming visit. National dairy herd improvement organisations provided animal and herd information, such as milk performance and culling data, and scoring of cows for lameness at regular milk performance tests for 99 herds. Appropriate key performance indicators for describing claw health in dairy cattle are the incidences of risk of lameness, 13 common claw lesions, and the annual culling risk directly related to claw and limb disorders. All data sets were for 2020. These key performance indicators were arranged in a benchmarking system using six classes (mean, median, 10th, 25th, 75th, and 90th percentiles) where farms in the 10th percentile represented the ‘best in class’.

**Abstract:**

While benchmarking is already used for the assessment of performance gaps in cattle herd management and welfare concerns, its application to quantifying claw health performance is relatively new. The goal here was to establish a benchmarking system for claw health in Austrian dairy cattle. We used electronically registered claw health data of cows from 512 dairy herds documented by professional hoof trimmers, culling data from the same herds, and locomotion scores taken at regular milk performance testings in 99 dairy herds during 2020. Mean, median and the 10th, 25th, 75th, and 90th percentiles of the incidences of risk of lameness, 13 common claw lesions, and the annual culling risk directly related to claw and limb disorders were used as key performance indicators. Only validated data sets were used and participating trimmers and locomotion scorers had to pass interobserver reliability tests with weighted Cohen’s kappa values ≥ 0.61 indicating substantial interobserver agreement. This claw health benchmarking system is intended to be used henceforth in the transnational cattle data network (RDV) by all participating farmers and is also available for veterinarians and consultants, with the agreement of respective farmers.

## 1. Introduction

Benchmarking today has applications in many modifications in industry, technology, service enterprises, public sectors and healthcare systems [1,2,3]. In principle, benchmarking describes the establishment of standards or reference points and is applied as a tool for competition analysis. It indicates the continuous comparison of products, services, processes and the methods of various ‘companies’ with the intention to reduce performance gaps to the ‘best in class’ systematically; that is ‘companies’ that demonstrate excellent process management. The basic idea of benchmarking is to assess the differences between ‘companies’ and establish why these differences exist, allowing for the identification of possible weaknesses and to optimise process workflows [1,2,3].

Benchmarking has now been introduced to cattle herd management. For instance, key figures have been established for dairy cattle welfare [4,5], dairy herd management [6], for analysing the effects of welfare standards (https://q-check.org/der-q-check-report; accessed on 14 March 2022) and biosecurity related to the administration of antimicrobials in beef fattening [7]. Further, benchmarks have been used to analyse the impacts of herd size and milk yield on cattle health [8], the effects of lameness, leg injuries, lying time, and facility design and management on cow comfort in dairy and beef cows across different countries [9,10,11].

The negative impact of lameness on milk yield, fertility traits, animal welfare and premature culling has been described by numerous researchers [12,13,14,15,16,17,18]. The basis for efficient monitoring of claw health in dairy cows is the electronic recording of regularly assessed claw health data obtained at each claw trimming visit. Their automated analysis using graphical charts illustrates various parameters, and the data can be stored by national breeding companies or other institutions [19,20,21]. Such centralized national registration of claw health data, regularly transmitted by professional hoof trimmers, has already been established in many countries [22,23,24]. In the Netherlands and Scandinavia, this has been long established for a very large percentage of dairy cows [22,25,26,27].

A benchmarking system in Sweden compares the claw health of approximately 66% of national dairy cows using regularly recorded data [26,28]. A similar tool has been implemented in the Danish SEGES herd management system [21,29]. Recently, key performance indicators for the prevalence of lameness, seven claw lesions and annual culling due to claw disorders have also been established in Switzerland [24,30,31].

A centralized, national registration of computerized claw health data was only established in Austria from 2017, even though many professional hoof trimmers had already used electronic documentation systems for their own purposes up until that time [19,32]. In 2017 the project *Klauen-Q-Wohl* was initiated by Rinderzucht AUSTRIA (www.rinderzucht.at; accessed on 14 March 2022) and ZuchtData EDV Dienstleistungen GmbH in cooperation with the Austrian Hoof Trimmers Association, numerous dairy farmers and other partners [33,34] using the technical infrastructure of the RDV [35]. The main objectives of this national project were to establish a nationwide infrastructure for centralized, standardized collection and analysis of electronically documented claw health data. These data can be used for genetic improvement, to provide tools for herd management and to establish a benchmarking system for claw health [33,34], which appears particularly suitable for implementation in a transnational cattle data network.

The cattle data network (RDV: *Rinder Daten Verbund*) is one of the largest cattle data networks in Europe. More than 54,000 farmers from Austria and the German federal states of Bavaria, Baden-Wuerttemberg, Nordrhein-Westfalen and Schleswig-Holstein, with more than 2 million cows, are registered [35]. Today this transnational database is tasked with the acquisition, storage and evaluation of herd health data and providing applications for livestock breeding, modern herd management and animal health services [35].

The aim of this study was to describe in detail the process of establishing a benchmarking system for claw health for Austrian dairy cattle using the centralized and standardized collection and analysis of electronically documented claw health data. This was recently realised within the framework of the national project *Klauen-Q-Wohl*. We aimed to address the incidence risk of lameness, the incidence risks of 13 claw lesions and the annual culling risk due to claw and limb disorders using documented claw health data from a large number of Austrian dairy herds. The greater goal of the project was to establish the basis for data processing so that this benchmarking system can be implemented as a future standard service for all RDV member farmers. This will provide them with a tool to compare the claw health performance of their own farms with other farms and to motivate them for ongoing improvement.

## 2. Materials and Methods

The basis for documentation of claw lesions by hoof trimmers was the harmonized terminology described in the ICAR Claw Health Atlas [36] and its Appendix 1 (Digital Dermatitis Stages—M stages) and Appendix 2 (Digital Dermatitis-Associated Claw Horn Lesions) [37,38]. The Atlas lists and illustrates 23 claw lesions [36]. However, notably, some of these lesions [39,40] and some digital dermatitis (DD) stages are not associated with pain [37,41]. For claw disorders that are always associated with pain and lameness, the term ‘alarm lesions’ was designated. These ‘alarm lesions’ include all ulcers (sole, toe, bulb ulcers), toe necroses, white line abscesses, all inflammatory swellings of the coronet and bulbs of the heel associated with deep digital sepsis, penetrating infected horn fissures, interdigital phlegmons, acute (M2) stages of DD, and all DD-associated claw horn lesions [42].

Benchmarking of claw lesion incidences was based on electronically registered claw health data. These were recorded by hoof trimmers participating in the *Klauen-Q-Wohl* project using the documentation program *Klauenmanager* (SEG Informationstechnik GmbH, Bad Ischl, Austria). This system is widely used by professional hoof trimmers in Austria and neighbouring countries [32,43]. It employs a modified 10 claw-zone system [44], whereby claw lesions can be recorded for each claw zone using three defined severity scores (mild, moderate, severe) [32]. All hoof trimmers in this project used professional hoof trimming boxes, i.e., hydraulic tilt tables that are very commonly used in Austria, or walk-in crushes. These trimming boxes were equipped with industrial electronic tablets with water-resistant and shockproof touch screens into which hoof trimmers continuously document observed claw lesions, and which provide access to claw disease histories for previously trimmed cows.

Claw health data recorded by 32 hoof trimmers at every trimming visit during 2020 from a total of 614 Austrian dairy herds were made available. The data had been acquired centrally by the Association of Austrian Cattle Breeders [33] using a serial interface. Further, in 55 of these dairy herds claw health data sets (566 claw lesion observations) were additionally recorded by the farmers themselves using the *Klauenprofi^®^* app, which was developed within the *Klauen-Q-Wohl* project [34,45]. The basis for documentation of claw lesions in this *Klauenprofi^®^* app is the same as previously mentioned for the documentation program *Klauenmanager.* Both use the terminology of the ICAR Claw Health Atlas [36] and describe six stages of digital dermatitis (M0, M1, M2, M3, M4, M4.1) [37,38]. This app does not use different severity scores for claw lesions. These claw health data sets were also submitted and collected using a serial interface by Rinderzucht AUSTRIA.

### 2.1. Definition of Key Performance Indicators for Claw Health and Benchmarking

Anonymised claw health data sets from participating hoof trimmers, farmers and LKV (Landes-Kontroll-Verband) employees were prepared by the data provider (ZuchtData) for the investigators. The latter made their evaluations and calculations and identified themselves merely by an assigned numerical code. For calculation of key performance indicators (KPI), only validated data sets were included following published guidelines, which, in particular, describe the validation of data recorded by hoof trimmers [20] and lameness data [46]. The particular parameters used for the validation of claw lesion data are described in Section 2.3.

In line with other authors [21,26,31,46], the following KPI were defined to be appropriate estimators of dairy cow claw health: the incidence risk of lameness, the incidence risk of various claw lesions, including the incidence risk of ‘alarm’ lesions that summarize all painful claw diseases, and the annual culling risk directly related to claw and limb disorders. These KPIs were then used to establish the claw health benchmarking system.

Subsequently, the KPIs were assigned to percentiles, notably the 10th, 25th, 50th (=median), 75th and 90th percentiles and the mean, as described by others [4,8,19]. This then allowed for comparison of individual farms with good (≤10th percentile), moderate (≥25th and <50th percentile) and poor (≥50th percentile) claw health.

### 2.2. Assessment and Evaluation of Lameness Data

Lameness benchmarking data were generated within the project *D4Dairy* (https://d4dairy.com/; accessed on 14 March 2022). Lameness and locomotion scores were contributed by 24 LKV employees who scored 7765 cows from 99 dairy herds that underwent regular milk performance testing every 30–40 days during 2020; again, within the scope of the *D4Dairy-Project*. For assessment of lameness, the five-point locomotion scoring method described by Sprecher et al. [12] was applied, such that score 1 indicates a non-lame cow. Over the observation period, 51,981 locomotion scorings were recorded from 99 herds. On average, 8.4 scorings per herd were carried out during 2020; however, approximately 6.7 scorings per cow were performed as there is some fluctuation in regard to the cow number per herd (replacements).

The 24 LKV employees had previously been trained in locomotion scoring for similar projects in recent years [13,14]. Additionally, these employees were retrained in locomotion scoring for the current project at a single commercial dairy farm by a single instructor in October 2019. Subsequently, the employees had to undergo an online interobserver reliability examination that presented various scenario videos of non-lame (score 1) and lame cows (scores 2–5). The details of the exam, including the technical setup and interobserver reliability practical test, were explained in advance to all participants at a single online joint meeting. Thereafter they had two weeks to complete the online video test.

For calculation of interobserver reliability (weighted Cohen’s Kappa values), two different approaches for ratings (locomotion scores) were used: firstly, the five-point locomotion scores were established, and secondly, these five-point scores were allocated to three categories, (i) combined scores 1 and 2 (non-lame and mildly lame), (ii) combined scores 4 and 5 (clearly and severely lame), and (iii) comprising score 3 (moderately lame).

For each locomotion scoring episode, the frequency of the different scores (1–5) was calculated and mean overall scores were used for benchmarking. The latter incorporated the lameness incidence risk (IR_Lame_) at cow level. This was calculated as the mean lameness incidence of all locomotion scorings taken during 2020 per herd and sorted into min (minimum), mean, median, 10th, 25th, 75th and 90th percentiles. As locomotion scoring was performed every 30–40 days at regular milk performance tests, each lame cow at these time points qualified as a ‘new’ case.

$${\mathrm{IR}}_{\mathrm{Lame}}=\frac{\mathrm{New}\;\mathrm{cases}\;\mathrm{of}\;\mathrm{lameness}\;\mathrm{in}\;\mathrm{cows}\;\mathrm{at}\;\mathrm{performance}\;\mathrm{testing}\;\mathrm{in}\;2020}{\mathrm{Total}\;\mathrm{number}\;\mathrm{of}\;\mathrm{cows}\;\mathrm{present}\;\mathrm{at}\;\mathrm{performance}\;\mathrm{testing}\;\mathrm{in}\;\mathrm{the}\;\mathrm{herd}\;\mathrm{in}\;2020}$$

### 2.3. Validation of Claw Lesion Data

Criteria for inclusion of centrally collected claw health data in this evaluation were as follows [20]:

- Only claw health data from Austrian dairy herds and from hoof trimmers that participated in the *Klauen-Q-Wohl* project were used,
- Only claw health data that passed the checks for animal ID plausibility, plausibility of recording date and diagnosis codes were used,
- Only data from dairy herds where at least 95% of data (de facto approximately 99%) were recorded by well-trained hoof trimmers were used,
- Only claw health data from 2020 were used, equating with only data recorded during the third year of this ongoing *Klauen-Q-Wohl* project. A minor component of the claw health data was recorded by farmers who participated in the *Klauen-Q-Wohl* project themselves using the newly developed documentation app *Klauenprofi*^®^ [34,45].
- Only data from dairy herds in which at least 50% of cows or more in respect to the mean cow number of the farm trimmed at the claw trimming visits (mainly two visits per year) were included, and data also had to include documentations from cattle without lesions, according to published guidelines [20,46]. The centrally acquired claw health data from these hoof trimmers and farmers had to contain at least five different claw lesions/diagnoses.

All hoof trimmers participating in the *Klauen-Q-Wohl* project were well-trained. The necessary requirement was the successful completion of a certified basic claw trimming course with 136 course units. Many of these hoof trimmers had also completed an advanced training course with another 80 units. Some of these participating trimmers passed a claw trimming instructor training course, the highest level of claw trimming education (https://www.klauenpflege.at/kurse; accessed on 14 March 2022). In all these training courses, participants were trained not only in the correct technique of claw trimming but also in the correct electronic documentation of lesions. All training courses had been organized in the same manner with the same contents by the LFI (Ländliches Fortbildungsinstitut [Continuing Education Institute of the Austrian Chamber of Agriculture]; https://www.lfi.at/; accessed on 14 March 2022) in cooperation with the Association of Austrian Hoof trimmers (AÖK; https://www.klauenpflege.at/; accessed on 14 March 2022). Further, at the outset of the *Klauen-Q-Wohl* project, all trimmers were equipped with the *Klauenmanager* program and participating farmers were equipped with the *Klauenprofi*^®^ app and additionally trained in using the correct terminology for claw lesions by author M.S., according to the ICAR Claw Health Atlas Codes [36,37,38] required for electronic documentation of these lesions.

The need for participating hoof trimmers to recognize claw lesions correctly was evaluated by an online interobserver reliability test using the SurveyMonkey^®^ program (SurveyMonkey^®^, San Mateo, CA, USA) at the end of 2019. This test assessed agreement (weighted Cohen’s Kappa values) across hoof trimmers with the correct diagnosis, whereby 50 pictures of various claw lesions (claw horn lesions and infectious claw diseases) were presented in the SurveyMonkey^®^ program. The pictures had been taken during multiple farm visits and at the clinic by author J.K. They represented all the claw lesions mentioned in the ICAR Claw Health Atlas [36] and Appendices 1 and 2 [37,38]. Each picture showed only one particular lesion, or, if this was not possible, the claw lesion in question was encircled. Clearly, with 50 selected pictures the same type of lesion (e.g., sole ulcer, white line disease, sole haemorrhage, M2 stage of DD) was presented more than once. We made sure that different pictures showed lesions from different feet or cattle and with different severity scores. However, in the online interobserver reliability exam, we did not ask participants to assign the three severity scores of claw lesions. The technical setup and the practical procedure of the interobserver reliability test were explained to all participants during one online joint meeting, and thereafter they had two weeks to complete the test.

In the interest of comparability of claw health data documented by hoof trimmers and farmers, the data were further processed. Thus, severity score 1 (mild) lesions of sole haemorrhage, double sole, white line disease (WLD) and heel horn erosion recorded by hoof trimmers were not included in the calculation of KPI. Only lesions of severity scores 2 (moderate) and 3 (severe) were used for further evaluation. In addition, all painful claw lesions were aggregated to ‘alarm’ lesions, including all ulcers, toe necroses, white line abscesses (WLA: severity scores 2 and 3 of WLD), inflammatory swellings of the coronet and bulbs of the heel associated with deep digital sepsis, interdigital phlegmons, penetrating and infected horn fissures, acute (M2) stages of DD, and all DD-associated claw horn lesions [42].

For benchmarking DD incidence risks, only those farms with an endemic DD infection were evaluated. Therefore, data of only 286 farms were included. Farms were considered to be DD-infected when at least more than two animals per farm with stages M1 to M4.1 of DD were recorded [37].

The KPI incidence risk of claw lesions (IR_ClawL_) was defined as the percentage of cattle with at least one documented claw lesion per mean cow number per year for 2020. Because hoof trimming visits to these farms occurred either twice or three times per year, i.e., at intervals of four to six months, each claw lesion was counted as a ‘new’ lesion in accordance with published reports [20,46].

$${\mathrm{IR}}_{\mathrm{ClawL}}=\frac{\mathrm{New}\;\mathrm{cases}\;\mathrm{of}\;a\;\mathrm{specific}\;\mathrm{claw}\;\mathrm{lesion}\;\mathrm{within}\;365\;\mathrm{days}}{\mathrm{Total}\;\left(\mathrm{mean}\right)\;\mathrm{number}\;\mathrm{of}\;\mathrm{cows}\;\mathrm{present}\;\mathrm{in}\;\mathrm{the}\;\mathrm{herd}\;\mathrm{within}\;365\;\mathrm{days}}$$

### 2.4. Assessment of Annual Culling Risk Due to Claw and Limb Disorders

Annual culling risk data due to claw and limb disorders, but also for a total of ten culling reasons, have been collected routinely from all dairy farms in Austria during regular performance testing for many years by LKV AUSTRIA. These are published annually in a report [47].

Culling data from the same dairy herds during 2020 were used for benchmarking the annual culling risk due to claw and limb disorder. In this respect, all ten dairy cow culling reasons for these farms for 2020 were assessed and summarized. From these data sets the percentage annual culling risk due to claw and limb disorders (ACR_Claw_) was estimated at the farm level. From these farm-specific prevalences, the mean, median, 10th, 25th, 75th and 90th percentiles were calculated.

$${\mathrm{ACR}}_{\mathrm{Claw}}=\frac{\mathrm{Number}\;\mathrm{of}\;\mathrm{cows}\;\mathrm{culled}\;\mathrm{due}\;\mathrm{to}\;\mathrm{claw}\;\&\;\mathrm{limb}\;\mathrm{disorders}\;\mathrm{within}\;365\;\mathrm{days}\;\mathrm{in}\;512\;\mathrm{dairy}\;\mathrm{herds}}{\mathrm{Total}\;\mathrm{number}\;\mathrm{of}\;\mathrm{cows}\;\mathrm{culled}\;\mathrm{within}\;365\;\mathrm{days}\;\mathrm{in}\;\mathrm{these}\;512\;\mathrm{dairy}\;\mathrm{herds}}$$

### 2.5. Statistical Analysis

Statistical analysis was performed using Microsoft Excel (Excel 2019^®^, Microsoft Corp., Redmond, WA, USA) and SPSS^®^ Statistics for Windows, version 25.0 (IBM Corp., Armonk, NY, USA). Mean, standard deviation, median, minimum values and percentiles were computed.

The weighted Cohen’s kappa value of the interobserver reliability for evaluation of the degree of agreement in the correct assignment of locomotion scores using the original five locomotion scores and three combined locomotion scores (combining 12/3/45) among the 24 LKV employees, and for evaluation of the degree of agreement in the correct assignment of 50 different claw lesions among the participating 32 hoof trimmers, were calculated using the package irr in R version 4.0.3 [48,49]. According to the criteria defined by Landis and Koch [50] and a recently published similar evaluation for claw health [24], a weighted Cohen’s kappa value of ≥0.61 was defined as the minimum requirement for including documented lameness and claw health data in the study. This kappa value indicates a substantial or even higher agreement across different operators [24].

After accurate data validation and exclusion of claw health data submitted from hoof trimmers and farmers that did not meet the selection criteria [20], and exclusion of the data of one hoof trimmer who did not complete the online test, the data sets of 512 herds from 31 hoof trimmers could be included in the evaluation. Statistical significance was defined as *p* < 0.05 against a two-sided null hypothesis of no difference.

## 3. Results

From the 512 dairy farms, where the claw lesion data from hoof trimmers and the annual culling data were available, 375 (73.2%) had free-stall systems and 137 (26.8%) tie stalls with at least 90 days access to an outside run. The mean herd size was 34.8 cows (range: 16–163), the mean 305-day milk production was 8581 kg (range: 4151–13,959 kg), and 70.1% of the cows were Austrian Fleckvieh, 18.7% Holstein and 10.3% Austrian Braunvieh cattle, and the rest were other breeds.

From the 99 dairy farms from which locomotion score data were available, 89 (89.9%) had free-stall systems equipped with automatic milking systems and 10 (10.1%) tie stalls with at least 90 days access to an outside run. The mean herd size was 61.8 cows (range: 26–132), the mean 305-day milk production was 8865 kg (range: 5232–12,165), and 81.9% of the cows were Austrian Fleckvieh, 12.0% Holstein and 5.7% Austrian Braunvieh cattle, and the rest were other breeds.

### 3.1. Interobserver Reliability for Locomotion Scoring

Results of the interobserver reliability tests of locomotion scoring from the 24 LKV employees that scored the 7765 cows in 99 dairy herds during one lactation in 2020 showed a mean weighted Cohen’s kappa value of 0.65 (min: 0.55; max: 0.75; median: 0.67), and 80% of the observers were above 0.6. Merging the original five locomotion scores into three categories combining scores 1 and 2 and scores 4 and 5 increased interobserver reliability substantially with a mean weighted Cohen’s kappa value of 0.72 (min: 0.61; max: 0.85; median: 0.73), and 100% of the values were above 0.6. Therefore, all 24 LKV employees surpassed the threshold ≥ 0.61 of the weighted Cohen’s kappa value, so that data from these 99 herds could be included in the evaluation.

### 3.2. Interobserver Reliability for Recogition of Claw Lesions

A mean weighted Cohen’s kappa value of 0.86 (min: 0.69; max: 0.97; median: 0.87) was calculated in the interobserver reliability test when separately evaluating all different claw lesions (e.g., WLD and WLA, diffuse and circumscribed sole haemorrhages as two different lesions, all M-stages as different lesions). In this first evaluation, the lowest weighted Cohen’s kappa value was 0.69, and the highest was 0.97. However, higher mean weighted Cohen’s kappa values of 0.89 (min: 0.70; max: 1.0; median: 0.91) were obtained when claw lesions of the same type (e.g., WLD and WLA in one group, sole haemorrhage diffuse and sole haemorrhage circumscribed in one group, all digital dermatitis stages M1 to M4 in one group) were aggregated. For both evaluations, all lesions separately and aggregated lesions of the same type, all participating hoof trimmers had a weighted Cohen’s kappa value of ≥0.69. When all different claw lesions were evaluated separately, or when claw lesions of the same type were aggregated, 74.1% and 88.9%, respectively, had a weighted Cohen’s kappa value > 0.8. For the present evaluation, validated claw health data from 512 herds from a total of 614 dairy herds, and the submitted corresponding data of all 31 hoof trimmers, were included.

### 3.3. Incidence Risk of Lameness

The mean lameness incidence risk (locomotion score: LSC ≥ 2) for all 7765 cows (mainly Austrian Fleckvieh, Holstein and Austrian Braunvieh) from these 99 dairy herds for 2020 was 38.3%, independent of the stage of lactation. Similar lameness incidence risks were also determined for dry cows and for cows during their first 100 days in milk (DIM) (Table 1).

### 3.4. Incidence Risks of Claw Lesions

The mean incidence risks of 13 claw lesions for 17,838 cows (mainly Austrian Fleckvieh, Holstein and Austrian Braunvieh) from 512 dairy farms documented in 2020 are listed in Table 2. There was a wide variation from 0.7% to 33.2% of animals affected by these different claw lesions. In the claw horn lesion group, sole haemorrhages, double soles, white line abscesses, ulcers (including sole, toe and bulb ulcers) and a concave dorsal wall (and other stages of laminitis) were assessed on average over a range from 8.1% to 18.1% of cattle. Infectious claw diseases were represented by a mean incidence risk of 0.8% for interdigital phlegmon and 33.2% for digital dermatitis (DD) at cow level. For evaluation of the mean incidence risk of DD, only farms with endemic DD-infection were considered. Overall, an average of 30.1% of cows had a painful ‘alarm’ lesion (Table 2). From available data sets, it could be calculated that 55.8% of all 512 herds were endemically infected with DD, whereas the other herds were DD-free.

### 3.5. Annual Culling Risk Due to Claw and Limb Disorders

The mean annual culling risk due to claw and limb disorders in the 512 investigated farms was 8.4% (±13.3; median: 0.0).

### 3.6. Benchmarking Lameness Incidences

Lameness incidence risk data showed a wide variation among the 99 investigated farms, even when all examined cows were compared with dry cows and those in their first 100 DIM. During 2020, of those farms of the 10th percentile, 16.2% of all lactating cows, 19.5% of dry cows and 18.6% of cows in their first 100 DIM had locomotion scores ≥ 2, whereas, for farms in the 90th percentile, the corresponding lameness incidence risks were 67.7%, 84.1%, and 74.4%, respectively (Table 3). A locomotion score ≥ 3 was observed in 4.4% of all lactating cows, in 12.4% of dry cows and in 7.1% of cows in their first 100 DIM in farms of the 10th percentile, whereas in farms of the 90th percentile the corresponding lameness incidence risks were 27.9%, 43.8%, and 32.2%, respectively (Table 3).

In respect of severe lameness in farms of the 10th percentile, 2.1% of all lactating cows, 9.5% of dry cows and 5.1% of cows in their first 100 DIM had locomotion scores ≥ 4, whereas in farms of the 90th percentile, the corresponding lameness incidence risks were 13.3%, 29.3% and 20.8%, respectively (Table 3).

### 3.7. Benchmarking Claw Lesion Incidences

The incidence risk of the 13 different claw lesions showed a wide variation between the 512 farms (Table 4). A painful ‘alarm’ lesion was observed on average in 30.1% of cows (median 25.9%) in 2020. However, in farms of the 10th percentile, only 6.3% of cows had ‘alarm’ lesions, whereas, in farms of the 90th percentile, 62.2% of cows had these painful disorders.

On average, 13.6% and 12.5% (median: 11.3%/9.9%) of cows had some type of ulcer or white line abscess, respectively. In farms of the 10th percentile, no cow had an ulcer or white line abscess. In farms of the 25th percentile, only 4.9% and 3.1% of cows had ulcers and white line abscesses, respectively. In contrast, in farms of the 90th percentile, 29.9% and 29.0% of cows had ulcers and white line abscesses during 2020 (Table 4).

The incidence of DD in farms of the 10th percentile during 2020 was only 5.4%, and 9.5% in farms of the 25th percentile, but this increased to 75.7% of cows in farms of the 90th percentile (Table 4).

### 3.8. Benchmarking Annual Culling Risk Due to Claw and Limb Disorders

In half of the 512 farms, no cows had to be culled in 2020 due to claw and limb disorders. The annual culling risk due to claw and limb disorders was 13.3% in farms of the 75th percentile and 24.4% in farms of the 90th percentile (Table 4). The mean incidence risk of painful ‘alarm’ lesions in 2020 was 25.2% (SD: 18.6%, median: 21.9%) in farms of the 10th to the 50th percentiles where no cows were culled due to claw and limb disorders. In farms of the 75th and higher percentiles, a mean of 36.7% (SD: 23.3%, median: 33.8%) of cows had ‘alarm’ lesions in respect of culling.

This benchmarking system is currently implemented as a new and additional standard tool for herd health management in the transnational cattle data network (RDV) in Austria and four federal states of Germany. By implementing this benchmarking system in 2022 for lameness, claw lesions and the annual culling risk due to claw and limb disorders in the RDV an additional column will be added to this table (as shown in Table 3 and Table 4), presenting the incidence risk of the individual member farm (MY FARM). In the near future, further sub-classification breakdowns of the cow population are planned, such as classes with similar herd sizes, similar breeds, herd milk yields or similar housing systems. The precondition for the implementation of additional subgroups in the benchmarking system for claw health within the RDV is that a large number of validated claw health data sets from a sufficient number of farms will be made available.

## 4. Discussion

To our knowledge, this is the first publication that describes the process for establishing a benchmarking system for claw health, including multiple parameters, in detail. Sandgren et al. [26] reported a similar process including lameness, culling data and a number of welfare parameters, but not claw lesion incidences. In Sweden and Denmark, benchmarking of claw health using claw lesion incidences has been implemented for many years, but the particular process for establishing the system has not been described [21,26,27,28,29]. In Switzerland, only set claw health goals are stated for several claw health indicators, similar to ours, but without a benchmark [24,30,31,53].

Claw health of dairy cows can be described using various key performance indicators obtained from different sources. Such sources include regularly determined lameness prevalences over a two-week interval [46,54], at the time of routine monthly milk performance testing [13,14], regularly registered veterinary diagnoses [55] and annually recorded culling reasons directly associated with claw and limb disorders [47]. Further important data sources are electronically recorded claw lesions assessed at each claw trimming visit [19,25,53] and regularly scored M-stages of digital dermatitis at two-week intervals in the milking parlour or during pen walks using a DD-app [56]. These comprehensive claw health data, now available in an electronic format, have become an indispensable requirement for monitoring claw health in herds and individual cows in modern dairy farming [27,57,58].

Data provided by hoof trimmers for 2020 were used because this was the third and final year of the *Klauen-Q-Wohl* project. At this stage, the participating hoof trimmers were already perfectly trained in the correct documentation of claw lesions, and data from numerous dairy farms were available compared to earlier years. The year 2020 was also a time when lameness data from numerous farms were available within the scope of the *D4Dairy* project. Lameness data were exclusively collected by trained LKV employees, never by hoof trimmers.

There is a broad international consensus that a mean annual lameness incidence risk ≤ 5% (counting locomotion scores ≥ 3) is the aim in well-managed dairy herds [59,60]. A mean lameness prevalence (counting locomotion scores ≥ 2) ≤10% should definitely be the maximum for a one-time assessment [31]. However, in a well-managed dairy herd, there should be no cows with locomotion scores ≥ 4 (severe lameness) [14,61]. The strict determination of such KPI with predefined maximum threshold values for prevalences of lameness, defined claw lesions and the annual culling risk due to claw disorders, as established in Switzerland [31], are in all likelihood topics for ongoing discussion.

In anticipation, establishing KPI for claw health using a benchmarking system with the classification of dairy farms using percentiles [51,52] of incidence risks of lameness, claw lesions and annual culling risk due to claw and limb disorders would appear more meaningful and flexible in practice [6,8,26]. Such a benchmarking system can be updated annually using data sets provided by hoof trimmers and other data sources [8,62]. Such a flexible benchmarking system does not only allow an annual update of KPI but allows for their adaptation to different parameters. These include different dairy breeds (Fleckvieh, Holstein, Braunvieh, Jersey, etc.), different performance levels (high and low milk yields), different husbandry systems (loose housing, tie stall, pasture based) [8,62], and various welfare criteria as well as for other special operational considerations [6,9,11]; but always allows for a comparison with the ‘best-in-class’ farms.

Computation of KPI for claw health should only be made using validated data sets following published guidelines, especially describing validation of claw health data [20,46]. This meant that the data from 102 farms had to be excluded from the present evaluation. However, the 31 participating hoof trimmers showed good interobserver agreement in accurately recognizing claw lesions so that their validated data could be included. Kappa values of ≥0.61, indicating a substantial strength of agreement [50] for interobserver reliability of hoof trimmers at correctly diagnosing different claw lesions, were also reported recently as a suitable quality assurance feature for claw health data [24,31].

Combining the original five locomotion scores into three categories, i.e., merging scores 1 and 2 and scores 4 and 5, resulted in a mean weighted kappa value of 0.72 and a minimum value of 0.61, indicating substantial agreement [50]. Moderate-to-substantial weighted kappa values of 0.38 to 0.78 for intraobserver agreement were reported in another study using the same five-point locomotion scoring system [63]. Other authors have described that intra- and interobserver agreement tests scores 2 and 3 were more difficult to differentiate consistently compared with other levels in the five-point scale and that the acceptance threshold for overall intra- and interobserver reliability (weighted kappa coefficient of > 0.6) was exceeded only for the two-point scale when the five scores were merged as (12)(345) or (123)(45) [64]. An almost perfect interobserver agreement between only two observers with a weighted kappa value of 0.84 was achieved for locomotion scoring of cows of 122 dairy herds using a five-point scale [9]. However, in the latter study, lameness was categorised as either clinical lameness (scores ≥ 3) or severe lameness (scores ≥ 4) [9], which might further explain the almost perfect agreement. Grouping scores 1 and 2 as ‘non-lame’ and scores 4 and 5 as ‘severely lame’ for further statistical analysis is also commonly applied by other authors [65,66]. In light of these reports, the results of this study with a minimum weighted kappa coefficient ≥ 0.61 for the interobserver reliability tests in locomotion scoring, and ≥ 0.69 in correctly diagnosing claw lesions, appear quite suitable. Such grouping of locomotion scores 1 (non-lame) and 2 (mildly lame) and scores 4 and 5, both indicating greatly and severely lame cows, is justified for statistical analysis when investigating the effect of lameness on fertility or milk yield [13,14,15,16]. However, at the individual cow level, identification of cows with score 2 (mild) lameness and their early and adequate treatment are essential prophylactic management measures to avoid subsequent development to moderate and severe lameness in the herd [14,16,46].

The mean annual lameness incidence risk (LSC ≥ 2) of the investigated cows in 99 dairy herds in 2020 was 38.2%, independent of the stage of lactation. This is higher than the 27.7% reported recently for cows within the period from parturition to conception in a population of 97 dairy herds in Austria for the year 2014–2015 [14]. Similar and even higher mean lameness prevalences of 33.6% to 63.0% were reported in 122 North American freestall dairies, with a considerable variation in lameness prevalence and facility design and management in these farms and in three different regions of Canada and the USA [9]. Again, there was a wide variation in lameness prevalences in 59 dairy farms in New Zealand, which ranged from 1.2% to 36.0% of cows per herd with a mean lameness prevalence of 8.1% using the four-point (0–3 score) DairyCo mobility scoring [67]. A similar low mean lameness prevalence of 14.8% was reported for 1449 dairy cows in 78 farms in Switzerland. However, while the latter were assessed only at one time point, there was again considerable variation across farms [68].

A striking finding in the present study is the rather high incidence risk of lameness, with locomotion scores ≥ 3, in dry cows and cows in their first 100 DIM in farms classified to the 75th and 90th percentiles; but also, at 5.1% to 19.5% in the best 10% of farms (10th percentile). Lame cows showing locomotion scores ≥ 3 during the critical time between the dry period and the first 100 DIM have a much higher risk for deteriorating fertility traits, reduced milk yield, BCS loss, ketosis and premature culling compared to non-lame or mildly lame cows [14,15,16,17,18]. Therefore, prevention or reduction in lameness in cows with scores ≥ 3 during the dry period and early lactation is anticipated to have beneficial effects on these and other parameters and welfare [14,17,23]. This can be achieved by checking all cows for lameness at dry-off and routine claw trimming, and treatment at dry-off and again at 40 to 60 DIM together with concurrent regular locomotion scoring at two-week intervals [14,16,46,69]. Conversely, an explanation for these rather high incidence risks of lameness in dry cows and cows in the first 100 DIM is that many farmers do not routinely check cows for lameness at dry-off and during the first 100 DIM, and do not perform hoof trimming at these critical time points [62,70]. In addition, regular and active monitoring of cows for lameness at two-week intervals, as recommended [46,54], is not yet implemented on many farms. In Austria, seasonal hoof trimming of the entire herd twice a year, in spring and autumn, with the exception of dry cows, is still widely performed [62,70].

In respect of the assessed lameness incidences of approximately 38.3% for these three cow groups, it must be taken into consideration that the mean number of lameness observations in dry cows was quite low, with 1.4 observations compared to 2.1 observations in cows in their first 100 DIM and approximately 6.7 observations for all lactating cows. This difference in the number of lameness examinations may also have an effect on the final calculated lameness incidence risk by raising the number of lame cows when assessment frequency increases [54]. The rather high incidence risks of lameness in dry cows could be associated, partly, with walking difficulty seen in heavily pregnant cows.

Risk factors for lameness in 30 and 80 Austrian dairy herds kept in cubicle loose housing systems have been evaluated in two former studies. These researchers reported that the most important risk factors included reduced lying comfort, inappropriate neck rail position, overstocking, slatted floors, a body condition score < 3.5 (for Austrian Fleckvieh cows), inadequate energy or protein supply indicated by a milk protein content < 3.2% or >3.8%, higher lactation number, no provisions for integration of heifers into the herd and no access to an outside run [65,66]. The reasons for the low or high incidence risks of lameness, claw lesions and annual culling risks in the present study were not investigated. This will be the subject of a follow-up study.

Similar incidences of claw lesions as determined in the present evaluation for 2020 in 512 Austrian dairy herds were reported in 686 Dutch [22] and 238 Swiss dairy herds. In the latter, there were distinctly lower prevalences of the concave dorsal wall, sole ulcer and DD [53]. Lower prevalences were also described in Canadian and Spanish dairy herds with 5.1%/8.8% for white line lesions, 6.4%/9.5% for sole ulcers and 21.4%/16.6% for DD, respectively [23,71]. However, a direct comparison of these results should always be made with caution, not least because of the inaccuracy of documentation itself, the proportion of cows trimmed and documented per herd per visit, the quantification method for claw lesions that were recorded several times per cow, and the rigour of data validation, all of which can have major effects on final prevalences [20]. In another evaluation that used similar hoof trimming data sets, significantly higher prevalences of sole haemorrhages and sole ulcers were detected during lactation months 1–4. Moreover, a higher prevalence of interdigital phlegmon around calving and the first two lactation months were reported [62]. All other claw lesions were fairly equally distributed over the lactation months and no significant clustering occurred at any particular lactation month [62].

In respect of animal welfare, this study highlights that attention should be focused on all painful claw lesions aggregated under the term ‘alarm’ lesion [42]. Since these lesions always cause pain, their prevalences ideally should be as low as possible. In contrast, higher prevalences of other claw lesions, not or only rarely associated with pain, such as heel horn erosion, concave dorsal wall contour (chronic laminitis), interdigital hyperplasia or sole haemorrhages, should not be interpreted as critical [31,32,40].

A focus on serious lesions was clearly important to other researchers as they decided to include only four, six or seven claw lesions in their benchmarking systems or as relevant KPI parameters for claw health. While there were only four in the Danish SEGES herd management system [21,29], there were six in the Swedish and Spanish Claw Health Online Systems [23,28], and seven in the Swiss system [24,31]. In the presented evaluation, 13 relevant claw lesions listed in the ICAR Claw Health Atlas [36] were included. We did not include usually non-painful score 1 lesions in the calculation of percentiles, mean and median. These are usually non-painful, mild lesions related to sole haemorrhages, double soles, white line separations and heel horn erosions.

It might be surprising for some readers to learn that in benchmarking digital dermatitis only 286 of the 512 farms could be included, and 226 farms had to be excluded from our evaluation. This was because these farms were free of DD. Consequently, the DD-herd prevalence was 55.8% for Austrian dairy herds. A very similar DD-herd prevalence of 49% was reported recently for Austria, underlining the actual situation in this country that, conversely, approximately half of dairy herds remain DD-free [70]. A survey performed in Austria approximately 14 years ago revealed a DD herd prevalence of only 15% [72]. This specific situation is in great contrast with the DD-infection status of herds in many other countries with a large dairy industry where more than 90% of dairy herds are endemically infected with DD [71,73].

The mean annual culling risk due to claw disorders in dairy cows in Switzerland was 11% in 2019 and 13.9% in 2020 [53]. This is substantially higher compared to an average of 7.4% in 2019 in registered dairy cows in Austria [47], and a mean of 8.5% in the present evaluation of 512 dairy farms in 2020. Swiss researchers defined <15% as the goal for the annual culling risk due to claw lesions in dairy cows [31]. When benchmarking this KPI, no cattle in these ‘best-in-class’ farms of the 10th to 50th percentiles were prematurely culled due to claw and limb disorders in 2020. This KPI reached 13.3% in farms of the 75th percentile and 24.4% in farms of the 90th percentile. Further, in farms of the 75th percentile or higher in respect of culling risk, the mean (median) incidence risk of ‘alarm’ lesions was 11.5% (11.9%) higher than in the farms up to the 50th percentiles where no cattle were culled due to claw and limb disorders. It can be assumed that cows on farms with higher culling risks were exposed to more risk factors that promote the development of ‘alarm’ lesions and therefore were prone to premature culling. However, painful claw lesions do not necessarily lead to premature culling. Outcomes always depend on how early and how effectively treatments are carried out, which can vary greatly from farm to farm [16,42,69].

Computation of KPI for claw health using a benchmarking system presents great opportunities to establish flexible reference values representing good claw health in ‘best-in-class’ farms and to compare this reference data with other classes of farms typifying moderate or poor claw health. Such benchmarking systems for claw health [21,26,29] and other herd health matters have also been described [4,6,8]. As Canadian researchers reported that benchmarking could motivate farmers and promoted cooperation between farmers and veterinarians to improve calf management and calf welfare [74,75], we expect a similar positive effect for Austrian farmers benchmarking claw health.

The use of claw health data provided by well-trained hoof trimmers equipped with electronic documentation systems and other trained personnel scoring cow locomotion regularly during milk performance testing is indispensable for the computation of KPI, and for determining herd prevalences for scientific evaluations [31,76,77]. Further, all of these data sets are of paramount importance for long-term genetic improvement of claw health in dairy cows [22,76,77,78]. Given the heritability estimates obtained in several genetic studies, claw health traits showed enough genetic variance to be included in the breeding program to select animals with less genetic susceptibility to claw lesions in the long term [22,76,77,78]. While the *Klauenmanager* program was used for evaluation in this benchmarking system, other documentation systems, e.g., *KLAUE* software (dsp-Agrosoft GmbH, Ketzin/Havel, Germany), can be used [24,31,53]. Periodical retraining of personnel in claw lesion diagnosis and locomotion scoring is advised to reach an acceptable level of interobserver reliability [9,24,31].

In addition, there is also a very practical application for centrally acquired claw health data in the RDV. The processed claw health data can be used for comparison by thousands of RDV-member dairy farmers. This benchmarking system for claw health is currently at the implementation stage in the RDV [35], with many participating dairy farmers in Austria and four federal states of Germany showing a similar farm structure and size as in Austria. In the RDV, benchmarking evaluation will be integrated into the already existing ‘LKV herdmanager’ program, which will enable a comparison of udder health, fertility traits and on-farm claw health, beginning in the first quarter of 2022.

## 5. Conclusions

The presented benchmarking system allows for the comparison of own farm claw health with a large number of other dairy farms matching similar performance levels, cow numbers and breeds through the cattle data network (RDV). The benchmarking of claw health may further support the analysis of improvement potential for the own farm, may encourage farmers and veterinarians to improve animal welfare and may be helpful for minimizing economic losses due to lame cows.

## Figures and Tables

**Table 1:** Mean incidence risks (%) of locomotion scores (LSC 1–5) of 7765 cows from 99 dairy herds for 2020 grouped into all lactating cows, dry cows and cows during their first 100 DIM (days in milk) with a total of 51,981 locomotion records; LSC 1: non-lame; LSC 2: slightly lame; LSC 3: moderately lame; LSC 4: clearly lame; LSC5: severely lame.

Cow Group	Number Cows	Number Scorings	LSC 1	LSC 2	LSC 3	LSC 4	LSC 5
All lactating cows	7716	45,900	61.2	24.2	8.8	4.2	1.0
Dry cows	4353	6081	60.6	24.8	9.1	4.1	1.3
Cows in first 100 DIM	6521	13,950	61.1	24.1	9.2	4.6	1.1
Total	7765	51,980	61.7	24.2	8.8	4.3	1.0

**Table 2:** Mean incidence risks of 13 claw lesions in cows from 512 dairy herds for 2020.

Type of Claw Lesion	%	SD
Thin soles	0.7	4.8
Interdigital phlegmon	0.8	2.0
Swelling of coronet and/or bulb	1.9	5.7
Horn fissure	2.6	4.5
Interdigital hyperplasia	5.0	7.3
Double sole	8.1	12.8
Corkscrew claw	8.3	13.9
Heel horn erosion	11.0	20.4
Sole haemorrhage (diffuse/circumscribed)	12.10	15.3
White line abscess	12.5	12.5
Ulcers (sole-, toe-, bulb-ulcers, toe necrosis)	13.6	11.5
Concave dorsal wall (+other laminitis stages)	18.1	18.5
Digital dermatitis	33.2	26.6
Alarm lesions *	30.1	21.5

* ‘Alarm’ lesions included all painful claw lesions—all ulcers, toe necroses, white line abscesses, interdigital phlegmons, swellings of the coronet and/or heel of the bulb, acute M2-stages of DD, and all DD-associated claw horn lesions); standard deviation (SD).

**Table 3:** Benchmarking lameness incidence at cow level per herd calculated as mean lameness incidence risk of all locomotion scores during one lactation period in 2020 with LSC ≥ 2, LSC ≥ 3 and LSC ≥ 4 by classification of the 99 herds using percentiles arranged into the min (minimum), mean, median, 10th, 25th, 75th and 90th percentiles; DIM: days in milk.

Percentage (%)		Percentiles					
Cows with LSC ≥ 2							
Annual Lameness Incidence	Min	Mean	10th *	25th *	Median	75th *	90th *
of all cows in lactation	8.0	39.4	16.2	24.6	37.0	48.8	67.7
of dry cows	9.2	46.2	19.5	28.7	43.3	56.8	84.1
of 100 DIM cows	11.3	40.7	18.6	26.8	36.1	49.7	74.4
**Percentage (%)**		**Percentiles**					
**Cows with LSC ≥ 3**							
Annual Lameness Incidence	Min	Mean	10th	25th	Median	75th	90th
of all cows in lactation	1.4	14.6	4.4	7.5	12.0	19.3	27.9
of dry cows	7.0	25.6	12.4	16.7	22.2	30.6	43.8
of 100 DIM cows	4.7	18.5	7.1	12.1	16.1	24.6	32.2
**Percentage (%)**		**Percentiles**					
**Cows with LSC ≥ 4**							
Annual Lameness Incidence	Min	Mean	10th	25th	Median	75th	90th
of all cows in lactation	1.0	6.5	2.1	3.2	5.0	7.9	13.3
of dry cows	4.5	19.8	9.5	12.2	17.9	24.5	29.3
of 100 DIM cows	2.7	11.8	5.1	7.6	10.2	13.9	20.8

* Interpretation of percentile classes: 10th percentile: 10% of all values are equal to or lower than this value and 90% of the values are above this value; each decile includes 10% of the data set, meaning that the first decile is the 10th percentile. 25th percentile: 25% of all values are equal to or lower than this value and 75% of all values are above this value; 50th percentile = median: 50% of all values are below and 50% are above this value; 75th percentile: 75% of all values are equal to or lower than this value and 25% are above this value; or in other words, the 25th percentile represents the median of all values below the 50th percentile, and the 75th percentile represents the median of values above the 50th percentile; 90th percentile: 90% of all values are equal to or lower than this value and 10% are above this value [51,52].

**Table 4:** Benchmarking claw lesion incidence risk and the annual culling risk due to claw and limb disorders (ACR_Claw_) of cattle from 512 Austrian dairy herds for 2020 by classification of the herds arranged into the mean, median and 10th, 25th, 75th and 90th percentiles.

Percentage of Cattle (%)	Code	Percentiles					
Key Performance Indicators		Mean	10th	25th	Median	75th	90th
**‘Alarm’ lesions**	ALARM	30.1	6.3	14.2	25.9	41.4	62.2
**Claw horn lesions and claw deformations**							
Thin sole	TS	0.7	0.0	0.0	0.0	0.0	0.0
Horn fissure	HF	2.6	0.0	0.0	0.0	4.2	8.5
Double sole	DS	8.1	0.0	0.0	4.7	10.3	18.5
Corkscrew claw	CC	8.3	0.0	0.0	2.9	9.6	25.9
Sole haemorrhage (diffuse/circumscribed)	SH	12.1	0.0	0.0	6.7	16.7	30.6
White line abscess	WLA	12.5	0.0	3.1	9.9	17.7	29.0
Ulcers (sole-, toe-, bulb-ulcers, toe necrosis)	UL	13.6	0.0	4.9	11.3	20.3	29.9
Concave dorsal wall (+other laminitis stages)	CD	18.1	0.0	4.9	12.9	26.8	41.4
**Infectious and interdigital lesions**							
Interdigital phlegmon	IP	0.8	0.0	0.0	0.0	0.0	3.0
Digital dermatitis	DD	33.2	5.4	9.5	25.4	52.2	75.7
Swelling of coronet/bulb	SW	1.9	0.0	0.0	0.0	0.0	4.9
Interdigital hyperplasia	IH	5.0	0.0	0.0	2.4	7.5	13.4
Heel horn erosion	HHE	11.0	0.0	0.0	1.4	10.9	37.8
**Annual culling risk due to claw and limb disorders**	ACR_Claw_	8.5	0.0	0.0	0.0	13.3	24.4

## Data Availability

Data is contained within the article.
